# Association between the detection of alcohol, illicit drugs and/or psychotropic medications/opioids in patients admitted due to trauma and trauma recidivism: A cohort study

**DOI:** 10.1371/journal.pone.0203963

**Published:** 2018-09-12

**Authors:** Sergio Cordovilla-Guardia, Celia García-Jiménez, Enrique Fernández-Mondéjar, Julián Fernando Calderón-Garcia, Fidel López-Espuela, Cristina Franco-Antonio, Sergio Rico-Martín, Pablo Lardelli-Claret

**Affiliations:** 1 Nursing Department, Nursing and Occupational Therapy College, University of Extremadura, Cáceres, Spain; 2 Department of Preventive Medicine and Public Health, School of Medicine, University of Granada, Granada, Spain; 3 Unidad de Cuidados Intensivos, Hospital Universitario Virgen de las Nieves, Granada, Spain; 4 Instituto de Investigación Biosanitaria IBS, Granada, Spain; 5 CIBER of Epidemiology and Public Health, Madrid, Spain; Monash University, AUSTRALIA

## Abstract

**Objective:**

To quantify the association between the presence and type of drug detected and trauma recidivism in a cohort of patients admitted due to trauma.

**Method:**

A cohort study was conducted based on data from a project where the presence of alcohol and other drugs (cannabis, cocaine, amphetamines, methamphetamines, tricyclic antidepressants, barbiturates, opiates and benzodiazepines) was analysed in 1,187 patients aged 16 to 70 years admitted due to trauma. The patients were followed for a period of between 10 to 52 months until June 2016. For this study, the recurrence of injuries from a sample of 929 patients from this cohort was analysed according to their consumption profile. Survival curves were estimated and adjusted Hazard Rate Ratios (aHRR) and adjusted incidence rate ratios (aIRR) were calculated.

**Results:**

The incidence rate of TR was 10.94 cases per 100 patient-years in the group of patients negative for substances and 27.99 per 100 patient-years in positive patients. The survival curves show very significant differences in cumulative recurrence-free survival between the groups (Log Rank: p<0.001). Both the aHRR and the aIRR estimates show an increased risk of re-injury due to alcohol consumption (aIRR: 2.33 (1.72–3.15), p<0.001), cannabis use (aIRR: 1.87 (1.09–3.20), p = 0.022) and polydrug use (aIRR: 2.34 (1.80–3.04), p<0.001).

**Conclusions:**

The presence of alcohol and/or illicit drugs in these patients doubles the risk of trauma recidivism.

## Introduction

There is little doubt about the role of alcohol as one of the main risk factors for trauma [1]. One study estimates that alcohol consumption be associated with a higher risk of trauma 5.7-fold [2]. There is also evidence in the literature of an increased risk of trauma associated with the consumption of illicit drugs such as cannabis, cocaine, amphetamines and other stimulants [3–6]. A similar increase was also found with the use of psychotropic medications/opioids with or without prescription, such as benzodiazepines or opiate derivatives [7]. Therefore, exposure to an increased risk of trauma from consumption of these substances could result in an increase in the history of injuries [8–10].

Trauma recidivism (TR) is defined as a chronic situation where different traumatic events appear on multiple occasions [11]. Previous studies have reported rates of TR ranging from 0.38% [12] in the general population to almost 90% [13] in studies performed in patients from substance misuse treatment centres. When we focus on those works that have addressed the issue of TR related to consumption, we found important methodological differences. Some of these studies only focus on certain types of injuries or mechanisms, such as traumatic brain injuries [14], intentional injuries [15], and traffic crashes [16], or only on severe trauma [17]. The periods of detection for recurrence also present important differences; studies have considered TR as any trauma prior to the trauma when consumption is detected [17], the five years previous to the trauma [9,18], and trauma that occurred in a follow-up of 5 years after the trauma when consumption was detected [16] and in follow-ups of 10 or more years [14,19]. However, the main element that prevents comparison among these works and, therefore, makes it difficult to derive conclusions regarding the association between consumption of various substances and TR is that these studies focus mainly on alcohol [10] with little attention to illicit drugs and psychotropic medications/opioids [18,19].

The reasons described above and the enormous socio-sanitary and economic impact of traumatic disease [20] justify the need to study the role of alcohol, illicit drugs and psychotropic medications/opioids in the risk of TR. Prospective longitudinal studies are the most reliable designs. However, they are expensive and involve inherent difficulties with long-term follow-up. This has led some authors to address this issue through an approach based on past trauma history [8,9,18]. Nevertheless, this approach faces important limitations. On the other hand, it is necessary to throw light on the magnitude of association between the consumption and TR takes into account the different consumption profiles and variables that could confound this relationship. Therefore, our objective was to quantify the association between the presence of these substances and the risk of TR in a cohort of patients admitted for trauma who were determined to have consumed alcohol, illicit drugs or psychotropic medications/opioids.

## Methods

### Study design and participants

A cohort study was performed based on data from the MOTIVA project [21]. This project aimed to implement a secondary prevention programme for alcohol and other drug-related injuries based on an SBIRT (Screening, Brief Intervention, and Referral to Treatment) [22] performed at the Virgen de las Nieves of Granada University Hospital of Traumatology (Spain). The MOTIVA project comprised a systematic screening for the presence of alcohol and other drugs in all patients between 16 and 70 years old who were admitted due to trauma and was active during the 31 nonconsecutive months: November 2011 to October 2012, June 2013 to November 2013, and June 2014 to June 2015. The research was approved by the Granada Provincial Research Ethics Committee (CEI-Granada) and conducted according to the principles expressed in the Declaration of Helsinki; including informed consent obtained from the participants or their relatives or guardians in minor participants.

**Fig 1:**
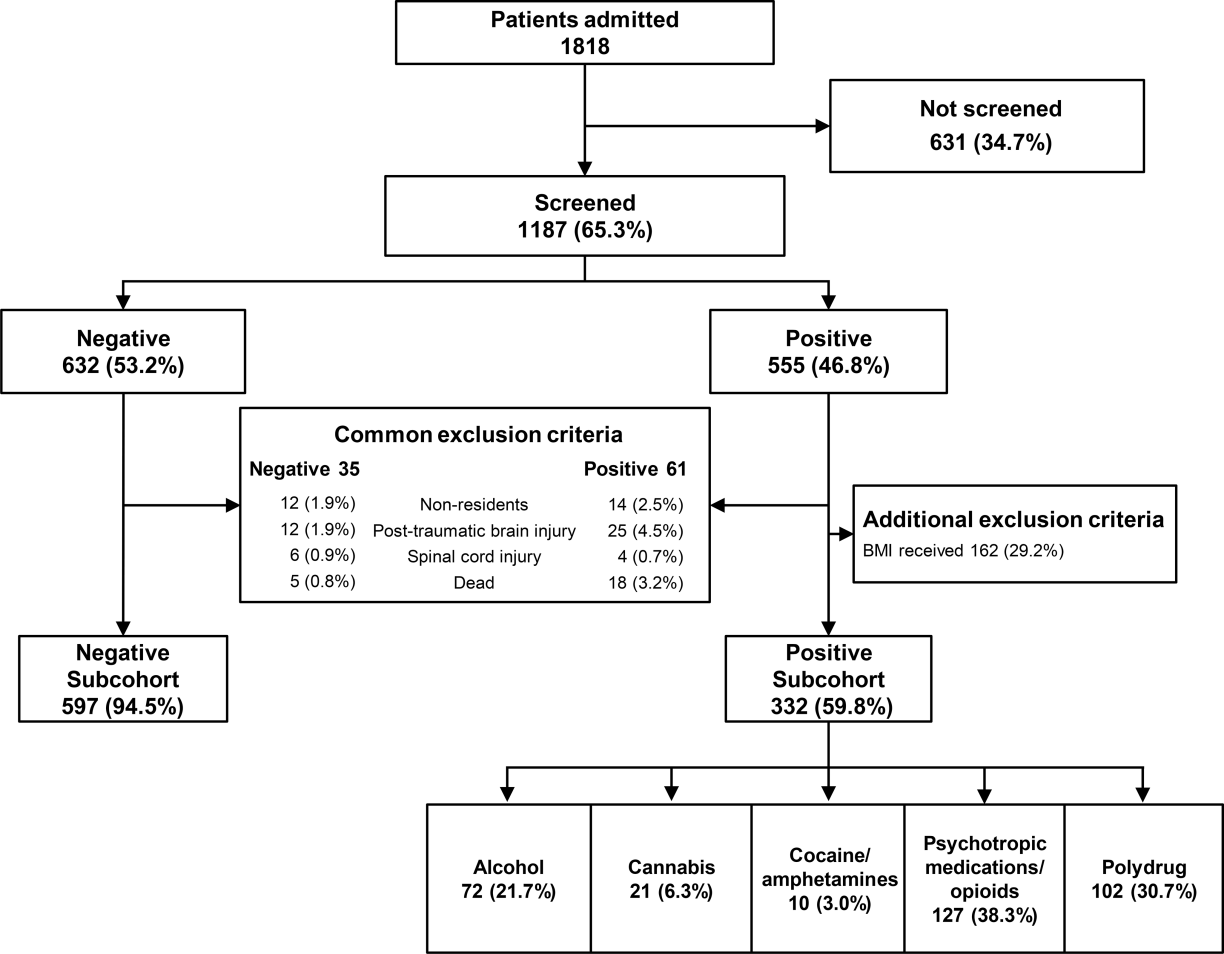
Flowchart of the distribution of patients. Patients admitted between 16 and 70 years old. BMI: Brief Motivational Intervention. Cocaine/amphetamines: positive for cocaine, amphetamines, and/or methamphetamines. Psychotropic medications/opioids, positive for benzodiazepines, tricyclic antidepressants, barbiturates, and/or prescribed opioids. Polydrug: positive for any combination of substances in the above groups.

The population eligible for the study was 1,818 patients hospitalized for traumatic injuries in the aforementioned hospital while the MOTIVA project remained active (Fig 1). Of the total number of patients, 1187 (65.3%) could be screened for alcohol and drug use (Characteristics of patients screened vs. not screened in S1 Table). Among the patients screened for substances, 632 (53.2%) had a negative result and the remainder (555, 48.4%) had a positive result for alcohol, illicit drugs and/or psychotropic medications/opioids. Of these, 258 patients were excluded from the study, either because they received a brief motivational intervention (BMI) in subjects with positive screening (162 subjects), or because they met the following exclusion criteria: non-residents, post-traumatic brain injury, spinal cord injury, or deceased. Once the exclusion criteria were applied, a sample of 929 subjects was divided into two subcohorts as follows: a negative subcohort (negative for alcohol, illicit drugs or psychotropic medications/opioids) with 597 patients; and a positive subcohort (positive for alcohol, illicit drugs and/or psychotropic medications/opioids) with 332 patients. The positive subcohort was divided according to the profile of consumption found into positive for alcohol (only alcohol consumption detected), positive for cannabis (only cannabis use detected), cocaine/amphetamines (positive for cocaine, amphetamines and/or methamphetamines), psychotropic medications/opioids (positive for benzodiazepines, prescription opiates, barbiturates and/or tricyclic antidepressants) and polydrug (positive for any combination of substances in the above groups).

### Baseline variables

Data for age, sex (male or female), mechanism of injury (traffic, sports, assault, falls on the same level, falls from a height, cuts or bruises, and other mechanisms), the Injury Severity Score calculated using Abbreviated Injury Scale Revision 2005 Update 2008 [23] and categorized into three levels (mild: 1 to 8, moderate: 9 to 15, and severe: ≥16), and past trauma history classified into three levels (nonrecidivist: first-time trauma patient, single recidivist: only one previous trauma, and multirecidivist: patients with more than one previous trauma) were collected prospectively during the patient's hospital stay by reviewing his or her electronic medical record. This record includes any medical care received by the patient in the Regional Health Service since 1999. Additionally, interviews with patients and their families were conducted.

### Definition of exposure

The SBIRT program was designed to carry out a systematic screening of exposure to alcohol and other drugs at the time of trauma patient admission to our hospital. Alcohol exposure was screened by blood testing and was considered positive when the blood alcohol level was higher than 0.03 g/mL. Screening for other drugs (cannabis, cocaine, amphetamines, methamphetamines, benzodiazepines, opiates, methadone, barbiturates or tricyclic antidepressants) was done with urine testing by fluorescence immunoassay. Review of the patient's medical records and direct questioning were used to distinguish between patients who screened positive for benzodiazepines and opioids as a result of emergency treatment of their injuries and patients who had taken these substances before they sought medical attention. Additionally, due to the different window of detection for each drug in urine, interviews were conducted to confirm whether the consumption occurred 6 hours before the injury. We ran a sensitivity analysis to determine the impact on the results of this self-reported 6-hour exposure cut-off.

### Follow-up and definition of outcome

The project involved following a dynamic cohort with follow-up times ranging from 10 to 52 months; follow-up for this study ended in June 2016 for the entire cohort of patients. Review of electronic health records and telephone calls were used to carry out the follow-up of patients. The digital medical records were consulted in the Regional Health Service Database. This database includes the patients’ medical history and records of any health care received at more than 1500 centres operated by the Andalusian Public Health Service. Trauma recidivism was defined as the occurrence of one or more new episodes of traumatic injury requiring medical care. The nurses who carried out the follow-up and collection of the data were blinded to patients’ exposure status. To detect deaths during follow-up, those that occurred in any health care facility were also searched for in the Regional Health Service Database. In addition, we consulted the database of the Provincial Institute of Forensic Medicine and funeral service records for the same period.

### Statistical analysis

For the descriptive part of the study, the baseline characteristics of the entire sample were compared (negative subcohort vs. positive subcohort) and according to the consumption profile for which hypothesis testing was used. A Chi square test was used for categorical variables, and due to the lack of a normal distribution, nonparametric tests were used for continuous variables (Mann-Whitney U and Kruskal-Wallis).

For the TR-free survival analysis during follow-up, Kaplan-Meier survival curves were calculated in the negative and positive subcohorts, and the curves were compared using log-rank test. To estimate the strength of association between each exposure category and the incidence of the first trauma during follow-up, adjusted Hazard Rate Ratios (aHRR) were estimated by including age, sex, mental disorders and history of previous traumas as covariates in the model due to their role as confounders (S1 Fig).

To consider not only the first trauma during follow-up but the total recurrence in each comparison group, incidence rate of TR (total number of traumatic injury events during follow-up) were estimated in each group, and adjusted incidence rate ratios (aIRR) were compared using a Poisson regression model with the same baseline covariates mentioned above.

In all of the models, the "negative" category was taken as the reference. For all estimates, the corresponding 95% confidence intervals (95% CI) were obtained. The results were considered significant at p<0.05. All analyses were performed using Stata Statistical Software, Release 14 (StataCorp, 2015, College Station, TX, USA).

## Results

When comparing the baseline characteristics between the positive and negative subcohorts (Table 1), we found differences in the distribution of injury mechanisms, with a higher proportion of traffic collision and sports injuries in the group of negative patients and a higher percentage of assault and falls in the group of positive patients. There were also significant differences in the presence of previously diagnosed psychiatric pathology, with a higher percentage in the group of positive patients (36.75%), and the distribution of previous history of trauma, with a more than two-fold increase in the proportion of multirecidivist in the group of positive patients compared to the negative subcohort (17.76% vs. 37.95%). We did not find significant differences in the other characteristics (age, sex and severity of trauma).

**Table 1:** Baseline characteristics of the sample (total, negative and positive to substances).

	Total (n = 929)	Negative Subcohort (n = 597)	Positive Subcohort (n = 332)	p
**Age (years)** Median (IQR)	44 (16–69)	44 (16–69)	45.5 (17–68)	0.182
**Sex** n (%)				
Male	612 (65.88)	395 (66.16)	217 (65.36)	0.805
**Mechanism of injury** n (%)				
Traffic collision	224 (24.11)	162 (27.14)	62 (18.67)	
Sports injury	71 (7.64)	63 (10.55)	8 (2.41)	
Assault	41 (4.41)	11 (1.84)	30 (9.04)	
Falls on the same level	307 (33.05)	187 (31.32)	120 (36.14)	<0.001
Falls from a height	126 (13.56)	77 (12.9)	49 (14.76)	
Cuts or bruises	108 (11.63)	69 (11.56)	39 (11.75)	
Other mechanisms	52 (5.6)	28 (4.69)	24 (7.23)	
**Injury Severity Score** n (%)				
Mild: 1 to 8	698 (75.13)	455 (76.21)	243 (73.19)	
Moderate: 9 to 15	161 (17.33)	104 (17.42)	57 (17.17)	0.192
Severe: ≥16	70 (7.53)	38 (6.37)	32 (9.64)	
**Mental disorders** n (%)	167 (17.98)	45 (7.54)	122 (36.75)	<0.001
**Past trauma history n (%)**				
Nonrecidivist	415 (44.67)	316 (52.93)	99 (29.82)	
Single recidivist	282 (30.36)	175 (29.31)	107 (32.23)	<0.001
Multirecidivist	232 (24.97)	106 (17.76)	126 (37.95)	

Note: IQR: Interquartile range; Single recidivist: Patients with only one previous trauma. Multirecidivist: Patients with more than one previous trauma.

The substances most frequently found were benzodiazepines (166 times, 17.87%), followed by alcohol (139, 14.96%), tricyclic antidepressants (70, 7.53%), cannabis (62, 6.67%), cocaine (35, 3.77%), opiates (22, 2.37%), barbiturates (8, 0.86%) and amphetamines (4, 0.43%). When grouping patients according to consumption profile, we found 72 (21.69%) positive for alcohol, 21 (6.32%) for cannabis, 10 (3.01%) for cocaine, amphetamine and/or methamphetamine, 127 (38.25%) for psychotropic medications/opioids (positive for benzodiazepines, tricyclic antidepressants, barbiturates and/or prescribed opioids) and 102 (30.72%) positive for any combination of substances in the above groups (Polydrug). In this last group, the substance that appeared more times combined was alcohol (65 times; 63.72% of patients in the group). In between-group comparisons of baseline characteristics (Table 2), age was greatest in the psychotropic medications/opioids group, with a median (Interquartile Range) of 54 (17–68) years. In contrast, cannabis users were younger with 25 (16–46) years. There were more men in all the consumption profiles except for the psychotropic medications/opioids group, where women were the majority. Differences were also seen in the mechanism of injury, highlighting that almost half of cannabis users had been in a traffic crashes, while more than half of the group using psychotropic medications/opioids had suffered a fall. The severity of the trauma, the history of mental disorders and the history of previous trauma also showed different distributions according to the profile, with a higher percentage of severe trauma (22.55%) and multirecidivist (48.04%) in the polydrug group and a much higher proportion of patients with mental disorders in the group consuming psychotropic medications/opioids (7 out of 10).

**Table 2:** Baseline characteristics of groups.

	Alcohol (n = 72)	Cannabis (n = 21)	Cocaine/ amphetamines (n = 10)	Psychotropic medications /opioids (n = 127)	Polydrug (n = 102)	p
**Age (years)** Median (IQR)	44.5 (17–67)	25 (16–46)	43 (25–53)	54 (17–68)	42 (20–68)	<0.001
**Sex** n (%)						
Male	66 (91.67)	20 (95.24)	8 (80.00)	41 (32.28)	82 (80.39)	<0.001
**Mechanism of injury** n (%)						
Traffic collision	15 (20.83)	10 (47.62)	2 (20)	11 (8.66)	24 (23.53)	
Sports injury	0 (0)	2 (9.52)	0 (0)	4 (3.15)	2 (1.96)	
Assault	10 (13.89)	2 (9.52)	0 (0)	4 (3.15)	14 (13.73)	
Falls on the same level	29 (40.28)	3 (14.29)	3 (30)	69 (54.33)	16 (15.69)	<0.001
Falls from a height	5 (6.94)	2 (9.52)	3 (30)	15 (11.81)	24 (23.53)	
Cuts or bruises	10 (13.89)	2 (9.52)	1 (10)	14 (11.02)	12 (11.76)	
Other mechanisms	3 (4.17)	0 (0)	1 (10)	10 (7.87)	10 (9.8)	
**Injury Severity Score** n (%)						
Mild: 1 to 8	54 (75)	15 (71.43)	9 (90)	99 (77.95)	66 (64.71)	
Moderate: 9 to 15	11 (15.28)	5 (23.81)	0 (0)	20 (15.75)	21 (20.59)	<0.001
Severe: ≥16	7 (9.72)	1 (4.76)	1 (10)	8 (6.3)	15 (14.71)	
**Mental disorders** n (%)	4 (5.56)	2 (9.52)	0 (0.00)	93 (73.23)	23 (22.55)	
**Past trauma history n (%)**						
Nonrecidivist	22 (30.56)	7 (33.33)	2 (20.00)	40 (31.50)	28 (27.45)	
Single recidivist	22 (30.56)	9 (42.86)	5 (50.00)	46 (36.22)	25 (24.51)	<0.001
Multirecidivist	28 (38.89)	5 (23.81)	3 (30.00)	41 (32.28)	49 (48.04)	

Note: N = 332. Cocaine/amphetamines: positive for cocaine, amphetamines, and/or methamphetamines. Psychotropic medications/opioids, positive for benzodiazepines, tricyclic antidepressants, barbiturates, and/or prescribed opioids. Polydrug: positive for any combination of substances in the above groups. IQR = interquartile range. Single recidivist: Patients with only one previous trauma. Multirecidivist: Patients with more than one previous trauma.

**Fig 2:**
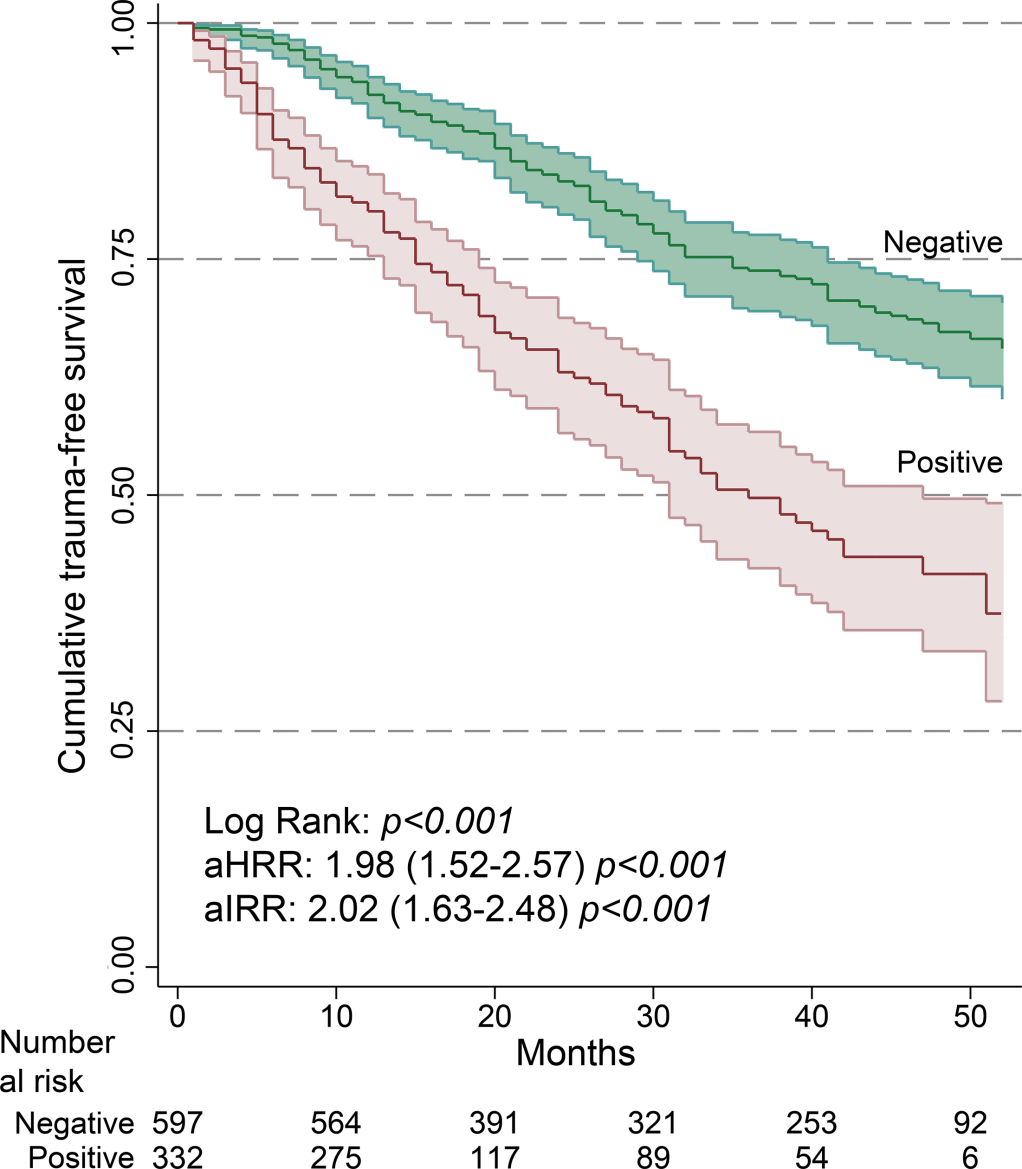
Kaplan–Meier curves of trauma-free survival in follow-up patients positive and negative for substances. Hall-Wellner bands represent 95% confidence intervals. aHRR: Hazard Rate Ratio adjusted by Cox proportional hazards regression. aIRR: Incidence Rate Ratio adjusted by Poisson regression. Covariates: age, sex, psychiatric disorder and past trauma history.

In the follow-up, we found that the median (Interquartile Range) of the months until the first trauma was 16 months shorter in the positive group compared to the negative group;16 (17–47) vs. 32 (13–31). The TR-free survival curves (Fig 2) show very significant differences between the subcohorts (Log Rank: p<0.001). These differences are maintained both in the model adjusted by Cox regression with a aHRR of 1.98 (1.52–2.57), p<0.001, and in the Poisson model with an aIRR of 2.02 (1.63–2.48), p<0.001.

The incidence rate of TR was 10.94 cases per 100 patient-years in the group of negative patients, and 27.99 cases per 100 patient-years when the patients in the positive subcohort were grouped for any substance, 31.14 cases per 100 patient-years if we consider the alcohol consumption group, 28.08 cases per 100 patient-years in the cannabis group, 24.74 cases per 100 patient-years in the cocaine/amphetamines group, 20.30 cases per 100 patient-years among consumers of psychotropic medications/opioids and 33.23 cases per 100 patient-years in the polydrug group.

The results of both multivariate regression models (Table 3) show an increased risk of recidivism associated with the detection of alcohol consumption (aIRR: 2.33 (1.72–3.15), p<0.001), cannabis use (aIRR: 1.87 (1.09–3.20), p = 0.022) and polydrug use (aIRR: 2.34 (1.80–3.04), p<0.001). Other variables that appear to be linked to an increase in the risk of new trauma during the follow-up period are mental disorders (aIRR: 1.45 (1.10–1.91), p = 0.009) and past trauma history (aIRR: 1.90 (1.47–2.45), p<0.001 for single recidivist (a single trauma prior to inclusion in the project) and aIRR: 2.46 (1.91–3.17), p<0.001 for multirecidivist). Results were not different in sensitivity analysis that included positive patients with self-reported of no exposure to substances 6 hours before the injury (S2 Table).

**Table 3:** Multivariate regression models.

	Cox proportional model aHRR (95%CI)	p	Poisson model aIRR (95%CI)	p
**Exposure**				
Negative	1.00 Ref.		1.00 Ref.	
Alcohol	1.90 (1.27–2.85)	0.002	2.33 (1.72–3.15)	<0.001
Cannabis	2.41 (1.25–4.64)	0.008	1.87 (1.09–3.20)	0.022
Cocaine/amphetamines	2.32 (0.95–5.68)	0.066	1.87 (0.83–4.26)	0.132
Psychotropic medications/opioids	1.36 (0.85–2.19)	0.202	1.21 (0.83–1.78)	0.327
Polydrug	2.28 (1.63–3.19)	<0.001	2.34 (1.80–3.04)	<0.001
**Age**				
1-year increase	0.99 (0.98–1.00)	0.057	0.99 (0.98–0.99)	0.018
**Sex**				
Female	1.00 Ref.		1.00 Ref.	
Male	1.04 (0.78–1.38)	0.805	0.96 (0.76–1.22)	0.740
**Mental disorders**				
No	1.00 Ref.		1.00 Ref.	
Yes	1.39 (0.97–1.97)	0.070	1.45 (1.10–1.91)	0.009
**Past trauma history**				
Nonrecidivist	1.00 Ref.		1.00 Ref.	
Single recidivist	1.72 (1.28–2.33)	<0.001	1.90 (1.47–2.45)	<0.001
Multirecidivist	2.45 (1.81–3.31)	<0.001	2.46 (1.91–3.17)	<0.001

aHRR: Adjusted hazard rate ratio using Cox proportional hazards regression. aIRR: Adjusted incidence rate ratio using Poisson regression.: First-time trauma patients. Cocaine/amphetamines: positive for cocaine, amphetamines, and/or methamphetamines. Psychotropic medications/opioids, positive for benzodiazepines, tricyclic antidepressants, barbiturates, and/or prescribed opioids. Polydrug: Positive for any combination of substances in the above groups. Single recidivist: Patients with only one previous trauma. Multirecidivist: Patients with more than one previous trauma.

## Discussion

From the results of this study, it can be deduced that the presence of alcohol or cannabis in patients hospitalized due to trauma was associated with an increased risk of TR. We also found an important increase in the risk of TR in the polydrug group. However, as alcohol was the substance most frequently found in different combinations with other drugs in the polydrug group (63.72% of patients were positive for alcohol in this group). This could suggest that the association between polydrug and TR was mainly due to this substance. Whether we take into account the first trauma during follow-up after discharge (almost 5 years), we found an increased risk of TR in all consumption profiles with the exception of the psychotropic medications/opioids and cocaine/amphetamines groups. However, the non-significant aHRR estimates in the latter group could be due to the small sample size: only 10 patients were positive screened for cocaine, amphetamines, and / or methamphetamines as a single substance. The consumption of these drugs often appears to be associated with other substances such as alcohol. Therefore, the majority of patients positive for cocaine, amphetamines, and / or methamphetamines were categorized in the polydrug group.

The associations found between the baseline characteristics of the patients and each subgroup of substances were also consistent with most of the previous studies. For example, the patients who tested positive for cannabis were young men (25 years, 95% men), and traffic crashes were the most common mechanism of injury [24]. In contrast, the characteristics of patients exposed to psychotropic medications/opioids differ from other substance users, as they were predominantly women, were older than the other groups, and had falls as the most common mechanism of injury. It is important to note that almost three quarters of patients in the psychotropic medications/opioids group had a history of psychiatric disorder. Given that the history of psychiatric disorders is a well-known risk factor for traumatic injury [18], the inclusion of this variable in the adjusted models is essential to establishing a relationship between the consumption of psychotropic medications/opioids and TR. The increase of 39%-45% in the risk TR that we found associated with mental disorders could be the reason for the absence of a significant risk of TR in the group of psychotropic drug users, despite the fact that in this group, the incidence rate was twice that of the negative subcohort.

In patients positive for the analysed substances, the time to new trauma in the follow-up period was 16 months; however, in the group of patients negative for all substances, the next trauma took twice as long to occur. The results of the adjusted models estimate an increase in risk attributable to the detection of alcohol from 90% in the case of the first new trauma to 133% if we take into account all traumas during follow-up. This strong association of alcohol consumption with TR has been shown in other studies [10,14,16]. In a systematic review, Nunn et al 2016 [10] found that the rate of alcohol-related trauma recidivism ranged from 26.7% to 76.9% among individual studies. They calculated a weighted aggregate estimate of 41.0%. However, this result should be interpreted with some caution due to methodological limitations and considerable variation among the studies included in this review. There may be considerable variations between studies due to the lack of a universally accepted definition for a trauma patient and disparity in the follow-up periods used to determine whether a repeat admission for injury had occurred. Studies also differed in the types of trauma patients they included. For example, Vaaramo et al 2008 [14] was to patients with injuries in a specific body region, such as the head. They found that even head trauma without traumatic brain injury under the influence of alcohol implies an elevated risk of subsequent traumatic brain injury (aHR 2.51, 95% CI 1.38–4.56, p < 0.01). Others have addressed this issue only in motor vehicle collision. With this approach, Fabbri et al 2005 [16] found that alcohol was the most powerful behavioural factor predicting recurrent events in subjects treated in an ED for injury after motor vehicle crash (relative risk: 3.73, 95% CI 3.00–4.64).

The presence of other substances in addition to alcohol is not usually contemplated. The studies that have addressed the relationship between the use of illicit drugs and TR find that cocaine is the substance that is most frequently associated with TR [9,19]. Those results are consistent with ours, if we consider the combined use of cocaine with other substances mentioned above. On the other hand, the increased risk of TR found in cannabis users is of particular relevance if we consider that it was the group with the highest proportion of road traffic crashes. Due to their potential to cause serious injuries, traffic crashes are a public health problem of the first order [20]. In recent years, an increase in cannabis use and a decrease in the perception of the risk of consumption for health in general have been detected in our country [25]. We also find a low perception when cannabis users who have suffered trauma are asked about the perceived risk that consumption entails at the time of suffering road traffic injury [26].

Our study shows that in addition to being common among trauma patients, exposure to alcohol is an important factor when detecting people at risk of new trauma. The clinical usefulness of the approach used in our study is that even if we ignore the history of consumption or its severity, the detection of exposure during systematic screening in patients admitted for trauma is sufficient to conclude that a patient has double the risk of suffering new trauma after discharge if no health education intervention is practiced. This reinforces the relevance of SBIRT programmes [22] in trauma centres and the BMI [23] as a tool to halve the risk of recurrence [21].

Our work has several strengths compared to other study designs. First, our study is based on a programme of systematic substance screening so that the possibility of selection bias in the original sample is lower than in other studies in which the detection of drugs depended on the decision of the physician [12,16]. Second, positive results for benzodiazepines and opiates have not been ruled out, but a distinction has been made between the patients who consumed these substances prior to the trauma and those to whom the substances were administered in the emergency room and by emergency services. This distinction has allowed us to quantify the effect of these substances on recurrence, unlike other similar studies [27,28]. Finally, the categorization of patients into subgroups according to consumption profile allows for a more valid assessment than those performed in other studies, where drugs other than alcohol were included in a single group [27–29].

The main limitation of this study is the confounding bias inherent in any observational study, which leads to some doubt in drawing conclusions regarding the causality of the associations observed. Although we have included the main factors related to both consumption and TR in the model, it is possible that there are other unmeasured confounders. Some candidates could be occupational factors or those related to the level of schooling [18]; however, cohort studies based on large populations of trauma patients [12,30] do not find a relationship between these variables and injuries in favour of other measures, such as young age, male sex or psychiatric disorders. Therefore, we consider that the potential bias of these confounders should not have much impact on our estimates. On the other hand, due to the different window of detection for each drug in urine, interviews were conducted to confirm whether the consumption of drugs other than alcohol occurred 6 hours before the injury. This may introduce social desirability and/or recall bias. However, this seems unlikely after revealing exposure to the substance with a laboratory test. The fact that the sensitivity analysis showed no differences supports this assertion. Another aspect to consider is that the intent of the injury has not been discriminated. However, often the intent is difficult to determine. We were able to collect information about diagnoses of mental disorder. Psychiatric conditions are strongly associated with self-harm, so we consider that an important part of the confusion related to intent is eliminated by introducing mental disorders as a covariate in the models. Finally, selection bias could arise because not all hospitalized trauma patients could be screened and subjects who had BMI were excluded. Nonetheless, we have no reasons to suspect an independent association between the presence of drugs and the probability of being screened or an association between the consumption profile and a higher probability of receiving the BMI.

## Conclusion

This study suggests that patients aged 16 to 70 years hospitalized for trauma who are screened positive for alcohol or cannabis have a higher risk of TR after hospital discharge. This study has also shown that the presence of multiple drugs doubles the risk of TR in these patients. Our results support the potential usefulness of programs for the systematic screening of alcohol and other drugs in trauma centres to performing to carry out activities of secondary prevention of injuries. Administrators and clinicians may optimize resources by knowing which patients are potential beneficiaries of these interventions or might benefit from more targeted or tailored interventions.

## Supporting information

S1 Data (DTA)

S1 Fig Graphical presentation of confounding using directed acyclic graph. (PDF)

S1 Table Characteristics of patients who were screened for substance consumption vs. patients who were not screened. (DOCX)

S2 Table Multivariate regression models without 6-hour exposure cut-off. (DOCX)
