# Supplementary material for: Association between the detection of alcohol, illicit drugs and/or psychotropic medications/opioids in patients admitted due to trauma and trauma recidivism: A cohort study
Source: PLoS One. 2018 Sep 12;13(9):e0203963. doi: 10.1371/journal.pone.0203963 (PMC6135508; doi:10.1371/journal.pone.0203963)
Supplement: S1 Fig — (PDF) [file pone.0203963.s002.pdf]

S1 Fig. Graphical presentation of confounding using Directed Acyclic Graph*

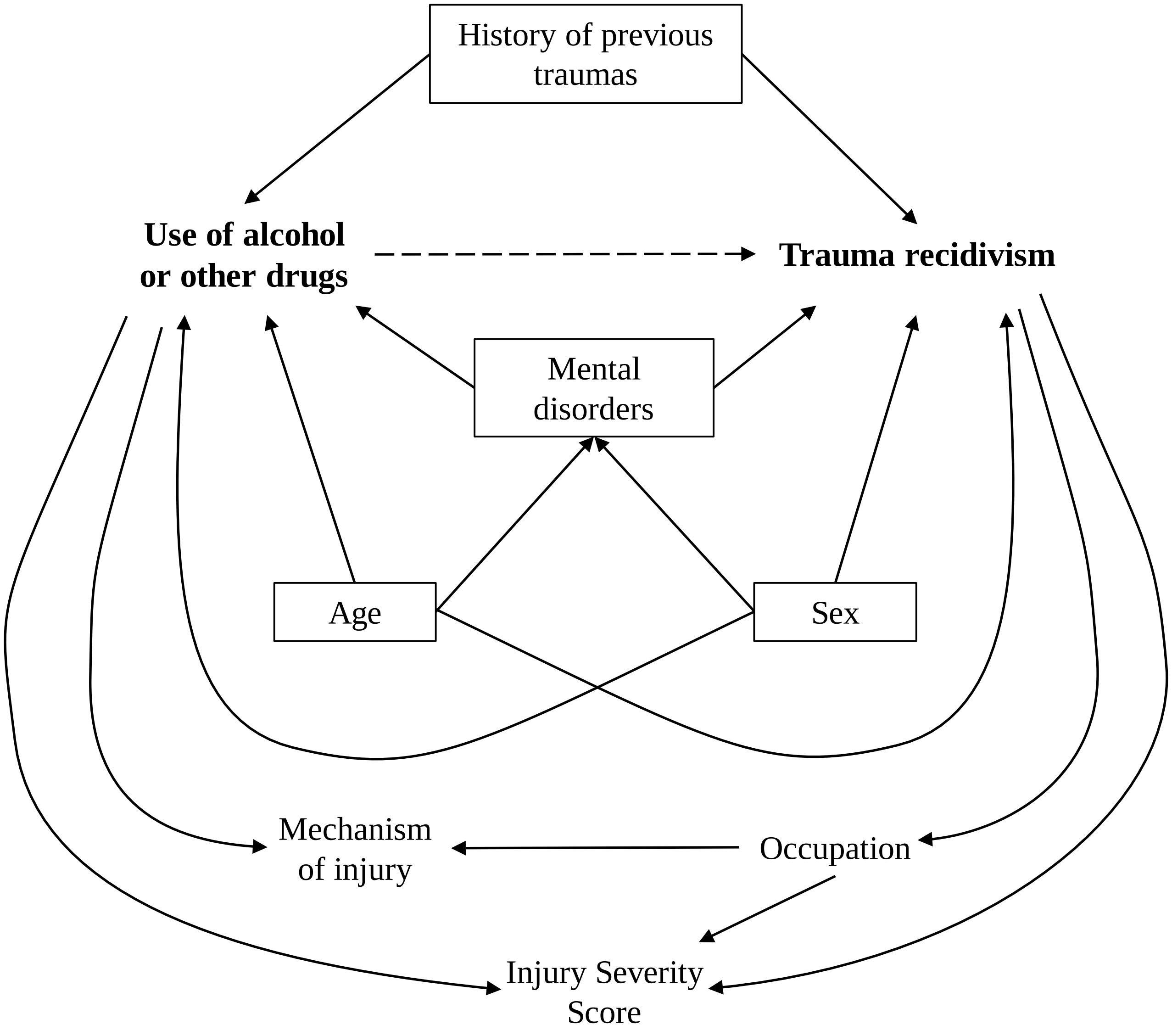

Note: To facilitate understanding of the graph, causal hypothesis relationships (arrows) were omitted between confounders (boxes) and non-confounders variables.

*Suttorp, M. M., Siegerink, B., Jager, K. J., Zoccali, C., & Dekker, F. W. (2015). Graphical presentation of confounding in directed acyclic graphs. Nephrology Dialysis Transplantation, 30(9), 1418-1423. http://doi.org/10.1093/ndt/gfu325

*Greenland S, Pearl J, Robins JM. Causal diagrams for epidemiologic research. Epidemiology. 1999 Jan;10(1):37-48
