# Supplementary material for: Association between the detection of alcohol, illicit drugs and/or psychotropic medications/opioids in patients admitted due to trauma and trauma recidivism: A cohort study
Source: PLoS One. 2018 Sep 12;13(9):e0203963. doi: 10.1371/journal.pone.0203963 (PMC6135508; doi:10.1371/journal.pone.0203963)
Supplement: S1 Table — (DOCX) [file pone.0203963.s003.docx]

**S1 Table**. Characteristics of patients who were screened for substance consumption vs. patients who were not screened

|  | Total  Admitted  (n= 1818) | Screened (n=1187) | Not Screened  (n= 631) |
| --- | --- | --- | --- |
| **Age (years)** Median [IQR] | 43 [30-54] | 43 [30-54] | 41 [30-53] |
| **Sex** n (%) |  |  |  |
| Male | 1244 (68.4) | 825 (69.5) | 479 (66.4) |
| **Mechanism of injury** n (%) |  |  |  |
| Traffic collision | 413 (22.7) | 316 (26.6) | 97 (15.4) |
| Sports injury | 147 (8.1) | 94 (7.9) | 53 (8.4) |
| Assault | 91 (5.0) | 64 (5.4) | 27 (4.3) |
| Falls on the same level | 600 (33.0) | 356 (30.0) | 244 (38.7) |
| Falls from a height | 225 (12.4) | 162 (13.6) | 63 (10.0) |
| Accidental cuts or bruises | 254 (14.0) | 127 (10.7) | 127 (20.1) |
| Other mechanisms | 88 (4.8) | 68 (5.7) | 20 (3.2) |
| **Injury Severity Score** n (%) |  |  |  |
| Mild: 1 to 8 | 1367 (75.2) | 822 (69.3) | 545 (86.4) |
| Moderate: 9 to 15 | 277 (15.2) | 217 (18.3) | 60 (9.5) |
| Severe: ≥16 | 174 (9.6) | 148 (12.5) | 26 (4.1) |
| **Deaths** n (%) | 31 (1.7) | 23 (1.9) | 8 (1.3) |

IQR: Interquartile range
