# Supplementary material for: Association between the detection of alcohol, illicit drugs and/or psychotropic medications/opioids in patients admitted due to trauma and trauma recidivism: A cohort study
Source: PLoS One. 2018 Sep 12;13(9):e0203963. doi: 10.1371/journal.pone.0203963 (PMC6135508; doi:10.1371/journal.pone.0203963)
Supplement: S2 Table — (DOCX) [file pone.0203963.s004.docx]

**S2 Table**. Multivariate regression models without 6-hour exposure cut-off

|  | Cox proportional model  aHRR (95%CI) | p | Poisson  model aIRR (95%CI) | p |
| --- | --- | --- | --- | --- |
| **Exposure** |  |  |  |  |
| Negative (n=575) | 1.00 Ref. |  | 1.00 Ref. |  |
| Alcohol (n=72) | 1.89 (1.29-2.78) | 0.001 | 2.45 (1.82-3.09) | <0.001 |
| Cannabis (n=26) | 2.59 (1.36-4.29) | 0.001 | 2.01 (1.51-3.04) | 0.001 |
| Cocaine/amphetamines (n=10) | 2.29 (0.94-5.78) | 0.070 | 1.79 (0.79-4.56) | 0.194 |
| Psychotropic medications/opioids (n=138) | 1.39 (0.86-2.09) | 0.182 | 1.25 (0.88-1.75) | 0.301 |
| Polydrug (n=108) | 2.88 (1.78-3.59) | <0.001 | 2.69 (1.98-3.56) | <0.001 |
| **Age** |  |  |  |  |
| 1-year increase | 0.99 (0.98-1.00) | 0.055 | 0.99 (0.98-0.99) | 0.017 |
| **Sex** |  |  |  |  |
| Female | 1.00 Ref. |  | 1.00 Ref. |  |
| Male | 1.06 (0.79-1.33) | 0.811 | 0.95 (0.77-1.12) | 0.801 |
| **Mental disorders** |  |  |  |  |
| No | 1.00 Ref. |  | 1.00 Ref. |  |
| Yes | 1.38 (0.97-1.96) | 0.075 | 1.46 (1.11-1.92) | 0.009 |
| **Past trauma history** |  |  |  |  |
| Nonrecidivist | 1.00 Ref. |  | 1.00 Ref. |  |
| Single recidivist | 1.62 (1.15-2.26) | <0.001 | 1.91 (1.51-2.38) | <0.001 |
| Multirecidivist | 2.54 (1.89-3.11) | <0.001 | 2.49 (1.95-3.21) | <0.001 |

aHRR: Adjusted hazard rate ratio using Cox proportional hazards regression. aIRR: Adjusted incidence rate ratio using Poisson regression.: First-time trauma patients. Cocaine/amphetamines: positive for cocaine, amphetamines, and/or methamphetamines. Psychotropic medications/opioids, positive for benzodiazepines, tricyclic antidepressants, barbiturates, and/or prescribed opioids. Polydrug: Positive for any combination of substances in the above groups. Single recidivist: Patients with only one previous trauma. Multirecidivist: Patients with more than one previous trauma
